# Skipping sarcoidosis: When ventricular arrhythmia arises immediately post–permanent pacemaker implantation

**DOI:** 10.1016/j.hrcr.2023.09.003

**Published:** 2023-09-11

**Authors:** Ekin C. Uzunoglu, Kevin Liu, David Spragg, Prakash G. Suryanarayana, Zoobia Z. Khan, Claude S. Elayi, John N. Catanzaro

**Affiliations:** ∗ University of Florida Health Science Center, Jacksonville, Florida; † Division of Cardiology, Johns Hopkins University School of Medicine, Baltimore, Maryland; ‡ Department of Pathology and Laboratory Medicine, University of Florida College of Medicine, Jacksonville, Florida; § CHI Saint Joseph Hospital – Cardiology, Lexington, Kentucky; ‖ Department of Cardiovascular Sciences, East Carolina University Health, East Carolina University, Greenville, North Carolina

**Keywords:** Sarcoidosis, Cardiac sarcoidosis, Implantable cardioverter-defibrillator, Permanent pacemaker implantation, Sudden cardiac death, Complete heart block, Ventricular tachycardia

**Key Teaching Points**

• Current guidelines suggest that select cardiac sarcoidosis (CS) patients with pacing indications undergo implantable cardioverter-defibrillator (ICD) implantation, given their increased risk of sudden cardiac death.
• The formal diagnosis of CS prior to urgent device implant for high-degree atrioventricular (AV) block would be optimal but is limited by the clinical instability, the timing, and the availability of the tools to obtain a timely formal diagnosis.
• Further research and discussion is needed regarding what level of evidence of CS is acceptable to implant an ICD directly instead of permanent pacemaker when formal CS cannot be established in a timely manner when a patient presents with potentially life-threatening AV conduction delays.

## Introduction

Sarcoidosis is a noncaseating granulomatous disease of unknown etiology. It is most often associated with pulmonary involvement but may also involve other organs and tissues, including the heart, whether it is symptomatic or not. Clinically manifest cardiac involvement occurs in about 5% of sarcoidosis patients, a rare and often unrecognized diagnosis, which may manifest in the forms of significant atrioventricular (AV) block, ventricular arrhythmia, and depressed ventricular function with or without heart failure at the time of diagnosis.^1^, ^2^, ^3^ Complete heart block (CHB) without obvious reversible causes typically requires urgent permanent pacemaker (PPM) implantation per the guidelines. However, current guidelines and latest studies suggest select cardiac sarcoidosis (CS) patients with pacing indications undergo implantable cardioverter-defibrillator (ICD) implantation owing to their increased risk of sudden cardiac death (SCD).^2^, ^3^, ^4^ If urgent pacing for heart block is needed among patients with suspected CS, a dilemma in choice of device type (PPM vs ICD) arises until formal CS is diagnosed.

We present a case to illustrate the dilemma between PPM vs ICD implantation in a patient with CHB and suspected CS.

## Case report

**Figure 1:**
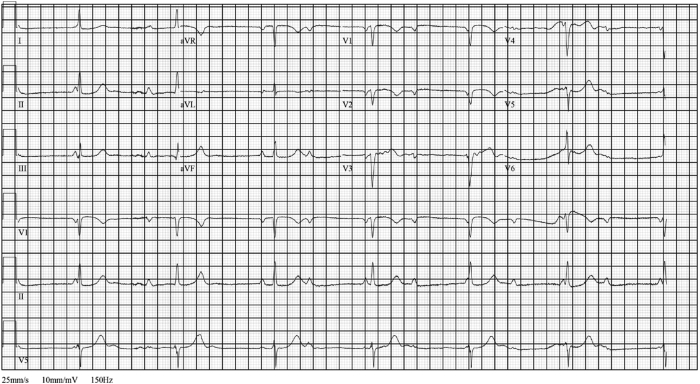
Standard 12-lead electrocardiogram demonstrated complete heart block with junctional escape.

**Figure 2:**
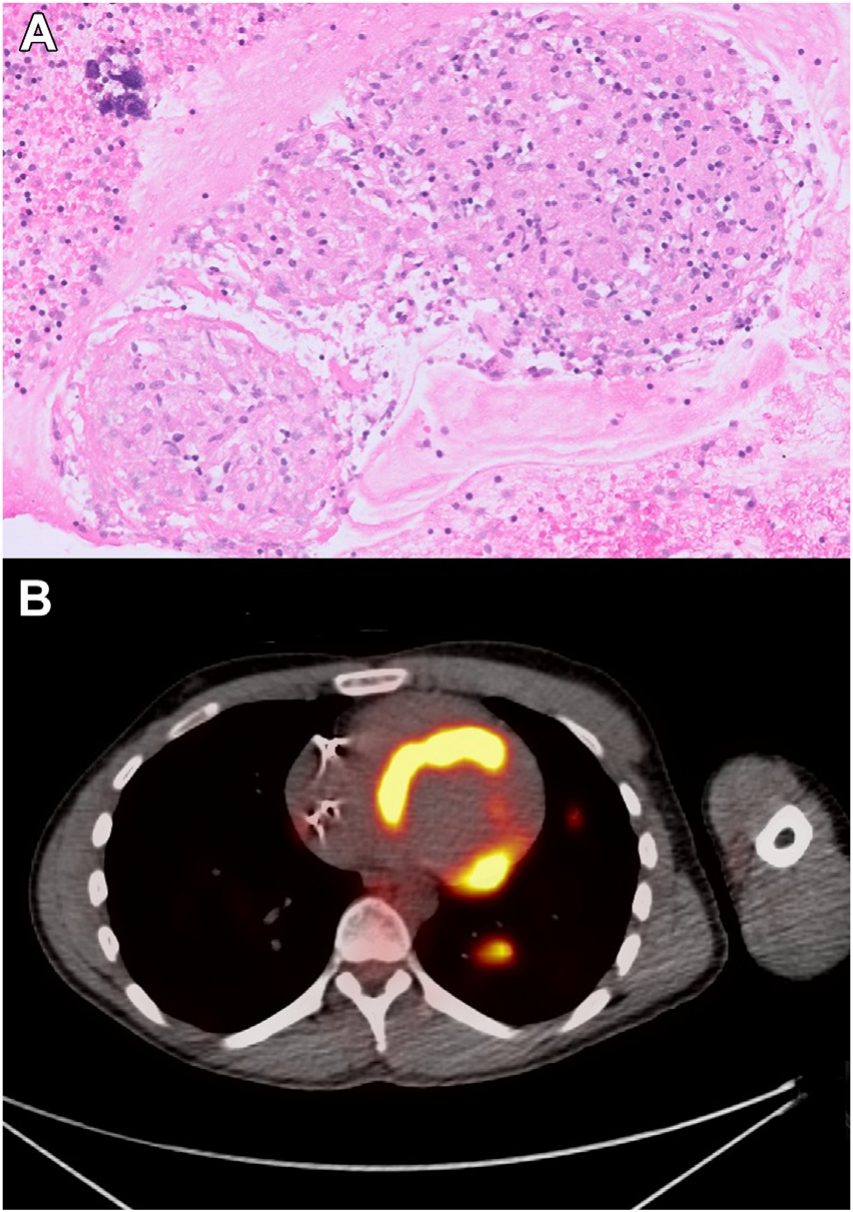
**A:** Subcarinal lymph node showing non-necrotizing granulomas and hyalinized fibrosis (hematoxylin-eosin, 20×). **B:** Fluorodeoxyglucose (FDG) – positron emission tomography image demonstrating FDG uptake in the basal lateral, inferior, and anterior walls with additional uptake in the right ventricle basal wall 1 week after discharge.

**Figure 3:**
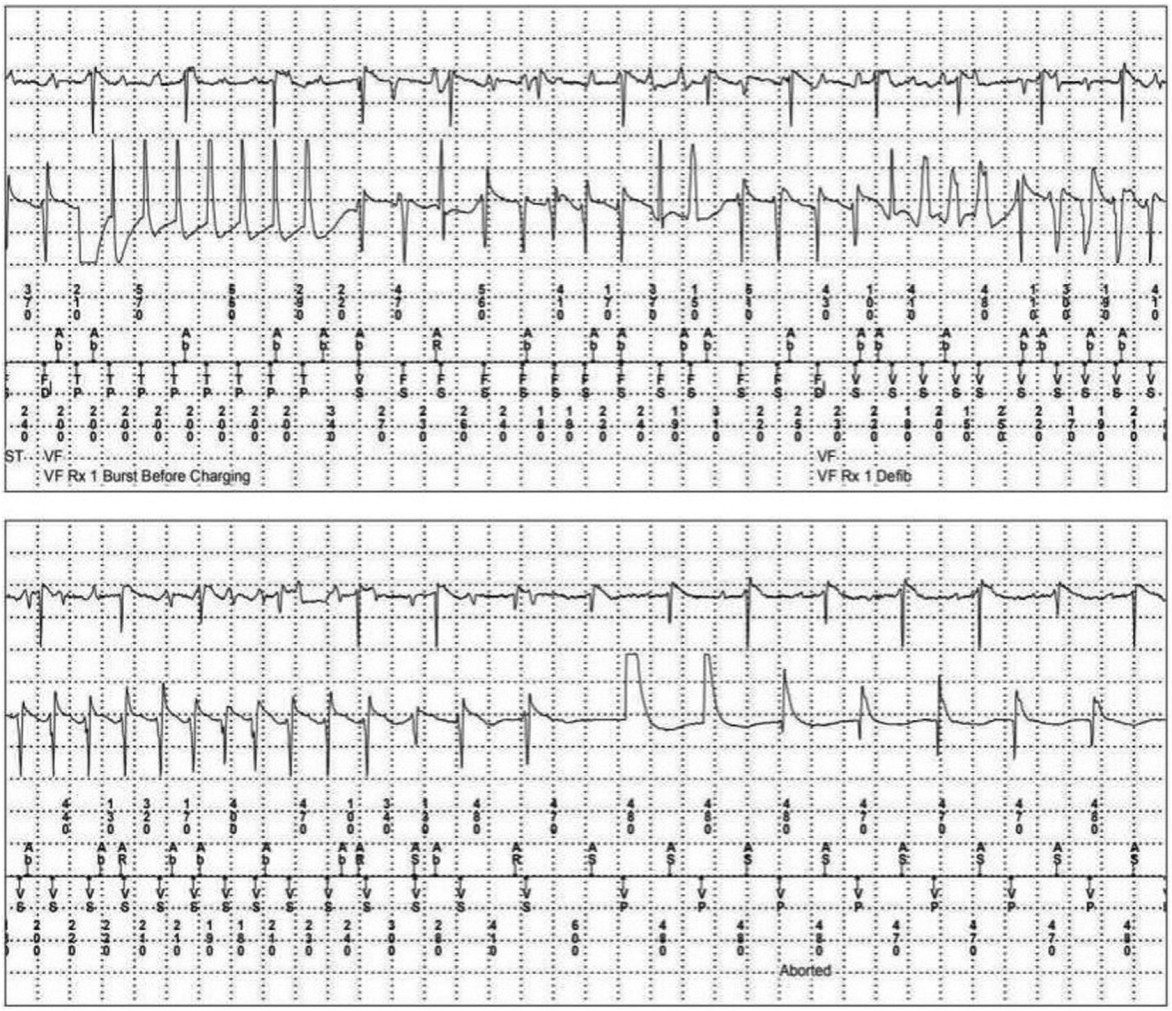
A polymorphic ventricular tachycardia fell in the VF zone and received 1 set of antitachycardia pacing (ATP) before charging. Conversion to sinus rhythm occurred a few seconds after ATP before a shock was delivered.

A 31-year-old male patient presented to the emergency department for dyspnea on exertion, palpitations, and lightheadedness worsening over the past weeks. Vitals were significant for bradycardia. Basic metabolic panel and the complete blood count were within normal limits. Initial standard 12-lead electrocardiogram showed CHB with junctional escape (Figure 1). Patient reported that the paternal uncle has a history of CHB in his 30s with sarcoidosis. The differential diagnosis included degenerative disease, infiltrative disease, Lyme disease, AV disease, tuberculosis, lymphoma, and endocarditis. There was no history of skin lesions, no recent trip to regions endemic for Lyme disease, and no B symptoms or enlarged nodes to favor lymphoma. Workup for other causes was unremarkable. Coronary computed tomography (CT) angiography showed patent coronary artery anatomy, diffuse pulmonary micronodules, multifocal splenic lesions, and hilar and mediastinal lymphadenopathies suggestive of possible sarcoidosis. Patient was admitted to the hospital and a chest radiograph indicated bilateral patchy interstitial opacities with correlative findings on cardiac CT. Echocardiography showed left ventricular ejection fraction of 55%–60% with normal wall motion. While undergoing a search for biopsy targets and pending cardiac magnetic resonance imaging (MRI, the patient became hemodynamically unstable and underwent urgent PPM implantation, precluding the ability to obtain a cardiac MRI. An electrophysiology study (EPS), which was considered, was not performed when the patient became unstable. After the PPM implantation, the patient was started on sotalol 80 mg twice a day, owing to symptomatic frequent premature ventricular contractions and runs of nonsustained ventricular tachycardia (NSVT). Subsequent sarcoidosis diagnosis was confirmed by endobronchial biopsy results 1 week after PPM implantation, with episodes of NSVT noted on telemetry in the meantime, warranting upgrade from PPM to ICD (Figure 2A). The sotalol dose was increased to 120 mg twice a day to further reduce the ventricular arrhythmia burden. The patient was discharged from the hospital on sotalol 120 mg in a stable condition. Cardiac fluorodeoxyglucose (FDG) – positron emission tomography (PET) demonstrated FDG uptake diffusely within the left ventricular septal wall, with a lesser degree in the basal lateral, inferior, and anterior walls, as well as the right ventricular basal wall, suggestive of CS (Figure 2B). The patient was started on methotrexate after PET scan as an outpatient by rheumatology. The patient continues to be totally pacemaker dependent, with 99.7% ventricular pacing requirements on device interrogation 3 months later. Device interrogation after 3 months demonstrated 5 NSVT events monitored; and device interrogation after 6 months demonstrated 129 NSVT and 1 ventricular tachycardia (VT) events monitored, and 2 polymorphic VT fell in the VF zone and received 1 set of antitachycardia pacing (ATP) before charging. Conversion to sinus rhythm occurred a few seconds after the ATP was delivered, so no shock was delivered (Figure 3).

## Discussion

It is imperative to confirm or exclude the diagnosis of CS in patients with CHB as early as possible owing to the increased risk of developing life-threatening ventricular arrhythmias and SCD.^1^^,^^2^^,^^5^^,^^6^ The current guidelines recommend pacemaker implantation in patients with CS with an indication for pacing even if the AV block reverses transiently.^1^^,^^2^^,^^4^ They also suggest ICD implantation can be useful in patients with CS and an indication for PPM implantations as a class IIA.^1^, ^2^, ^3^, ^4^ Two recent Finnish studies strongly reinforce the need for an ICD among patients with CS and advanced conduction system disease.^6^, ^7^ The rate of SCD / sustained VT was high (30.8%) in CS patients with advanced conduction disease system during a median follow-up of 4.1 years, even in patients with normal ejection fraction (5%).^6^, ^7^, ^8^

The Heart Rhythm Society (HRS) 2014 guidelines^2^ define 2 pathways to diagnose CS: firstly, histological diagnosis from myocardial tissue; and secondly, clinical diagnosis from invasive and noninvasive studies. Although guided endomyocardial biopsy demonstrating noncaseating granuloma on histological examination is one option to diagnose a patient with CS, it is generally not the first choice of diagnosis method owing to its low sensitivity and high procedural risks.^2^ The clinical diagnosis of CS is also complicated, as it consists of histological diagnosis of extracardiac sarcoidosis and exclusion of other causes for the cardiac pathology, in addition to the presence of at least 1 of the clinical presentations and/or 1 of the cardiac imaging modalities (cardiac MRI and FDG-PET).^1^^,^^2^ The Japanese Circulation Society published a 2017 guideline for diagnosis criteria of CS^9^ that did not mandate biopsy tissue confirmation (in contrast to HRS guidelines) when the patient shows clinical findings strongly suggestive of pulmonary or ophthalmic sarcoidosis with various imaging (abnormal PET, late gadolinium enhancement – cardiac MRI) or characteristic laboratory findings of sarcoidosis, and when clinical findings strongly suggest cardiac involvement. It remains an unresolved question whether CS can be diagnosed without a positive biopsy, as cardiac MRI is not specific of CS.^9^^,^^10^

In daily practice, the decisions leading to PPM vs ICD implantation may not be so straightforward. The advantages of directly proceeding with an ICD are to avoid exposing the patient to undue additional procedural risk (initial PPM implantation, followed by an upgrade to ICD) and to allow for protection against arrhythmia during diagnostic workup. On the other hand, the disadvantages of an ICD are increased risks of complications when compared to PPM, such as the additional risk of inappropriate shocks, as well as increased unnecessary costs if the patient turns out not to have sarcoidosis upon further diagnostic testing.^11^^,^^12^ Temporary pacing until there is a definitive diagnosis can be an option, as demonstrated in a retrospective study where 17 patients with CHB underwent a cardiac MRI with an active fixation pacing lead connected to a reusable extracorporeal pacing generator (temporary PPM) before the PPM implant.^13^ They have demonstrated that cardiac MRI in patients with a temporary PPM was safe and effective.^13^ However, temporary ventricular pacing may not always be stable enough to perform the necessary diagnostic procedures in unstable CHB. Another challenge is that cardiac MRI may not be easily available or even not available at all in a large number of practices. Finally, temporary pacing until a diagnosis is made may keep the patient in the hospital longer.

Another tool to risk-stratify patients with CS and CHB is the EPS, as recommended in the 2017 AHA/ACC/HRS guideline for management of patients with ventricular arrhythmias and the prevention of SCD.^3^ There is a class 2A indication for an EPS for patients like the one presented here when the diagnosis of CS is confirmed. We did consider doing an EPS to further decide between a pacer and an ICD, as a negative EPS provides a good negative predictive value for future ventricular arrhythmias and SCD.^3^^,^^14^ However, the patient became clinically unstable and underwent an urgent pacemaker placement.

What level of evidence of CS is acceptable to implant an ICD directly instead of PPM when formal CS cannot be established in a timely manner when a patient presents with potentially life-threatening AV conduction delays? A biopsy available with results obtained in a timely manner is certainly the gold standard but is rarely possible. A cardiac MRI with findings compatible with CS in a clinical setting suggestive of sarcoidosis might be acceptable for the diagnosis of CS if the MRI is feasible in a timely manner. If a cardiac MRI is not possible, it is unclear whether a chest CT, more widely and easily available, suggestive of pulmonary sarcoidosis would be enough to attribute high-degree AV block to CS. An EPS can also be considered when the likelihood of CS is high, as discussed above. As technology continues to progress, more options may become available. However, for now, we emphasize the importance of device management on a case-to-case basis.

## Conclusion

An incomplete diagnosis complicates optimal treatment algorithms and requires action in urgent clinical scenarios. In the case of CS, cardiac manifestations can include both high-degree AV block and ventricular arrhythmia, which may require treatment prior to a definitive diagnosis. HRS expert consensus gives a class IIa recommendation for ICD implantation in patients with CS with an indication for PPM implantation, which may suggest initial ICD implantation in even suspected CS patients. However, ICDs have been associated with a slightly increased risk of complications compared with PPMs and are more expensive. The formal diagnosis of CS prior to urgent device implant for high-degree AV block would be optimal, but this option is limited by the clinical instability, the timing, and the availability of the tools to obtain a timely formal diagnosis. Further research and discussion regarding options and treatment choices until the diagnosis need further exploration.
